# Incidence of vitiligo in children with Graves' disease and Hashimoto's thyroiditis

**DOI:** 10.1186/1687-9856-2011-18

**Published:** 2011-11-18

**Authors:** Brea Prindaville, Scott A Rivkees

**Affiliations:** 1 Yale Pediatric Thyroid Center, Department of Pediatrics, Yale University, New Haven, CT, USA

## Abstract

**Background:**

Limited data are available on the association between vitiligo and autoimmune thyroid disease in pediatric patients. In addition, reported studies of pediatric patients have been based on a population known to have vitiligo and subsequently evaluated for the presence of thyroid function abnormalities.

**Methods:**

A retrospective chart review was performed on 333 children who had been followed for thyroid disorders by endocrinologists at the Yale Pediatric Thyroid Center over the last 5 years for autoimmune thyroid disease. Demographical and clinical features of patients found to have thyroid disease and vitiligo were recorded. These studies were approved by the Yale Human Investigation Committee.

**Results:**

Of the total 333 children and adolescents, 9 (2.7%) were noted to have vitiligo. Four patients (44%) had Graves' disease and 5 patients (66%) had Hashimoto's thyroiditis. For patients with Graves' disease and vitiligo, the average age of onset of thyroid disease was young at 4 ± 0.7 years, and the diagnosis of vitiligo usually preceded that of thyroid disease. For children with Hashimoto's thyroiditis and vitiligo, thyroid disease was diagnosed at an average age of 13.25 ± 2.8 years.

**Conclusion:**

In our population, 4.6% of children with Graves' disease and 2.0% of children with Hashimoto's thyroiditis had vitiligo. Interestingly, when vitiligo presents with Graves' disease, it occurs in younger rather than older children.

## Introduction

The association between vitiligo and autoimmune thyroid disease, especially Hashimoto's thyroiditis, has been characterized in adults [1-3]. Studies indicate an autoimmune etiology for vitiligo, and genes have been identified that cause both vitiligo and autoimmune thyroid disease [1-11]. However, little data are available on the association of vitiligo and thyroid disease in children [1-3]. In addition, reported studies of vitiligo and thyroid disease are based on studies of children who were identified as having vitiligo and subsequently evaluated for the presence of thyroid function abnormalities [1-7].

Graves' disease is an autoimmune condition with an estimated incidence of 1 in 10,000 children [12,13]. Graves' disease most commonly occurs in teenagers, and less than 5% of pediatric patients present at less than 5 years of age [14,15]. Hashimoto's thyroiditis is also an autoimmune disease associated with the presence of anti-thyroid antibodies that can result in either hypothyroidism or a euthyroid state [16,17]. Like Graves' disease, the peak prevalence is in the teenage years [14,15,18,19]. The incidence of antithyroid antibodies in the pediatric population is as high as 1% in some studies [16-20].

Both Graves' disease and Hashimoto's thyroiditis are associated with other autoimmune conditions including diabetes mellitus, inflammatory bowel disease, celiac disease, autoimmune hepatitis, adrenal insufficiency, and vitiligo [14,17-20]. To better characterize the association between vitiligo and thyroid disease in pediatric patients, we performed a retrospective analysis of patients diagnosed with thyroid disease. We now report the prevalence and clinical characteristics of children with vitiligo in the setting of Graves' disease and Hashimoto's thyroiditis.

## Methods

A retrospective chart review was performed on 333 children who had been evaluated for autoimmune thyroid disorders at the Yale Pediatric Thyroid Center over the last 5 years. Patient ages ranged from 1 to 21 years. Standard clinical diagnostic criteria were used to establish the diagnosis of Graves' disease or Hashimoto's thyroiditis [21]. As part of routine clinical evaluation, all children cared for at the Yale Pediatric Thyroid Center are evaluated for eye disease, liver disease, thyroid disease, and vitiligo. Vitiligo was diagnosed by standard criteria [4], and confirmed by a dermatologist. In the patients noted to have vitiligo, demographical and clinical features of the thyroid disease were recorded. The age at diagnosis of vitiligo and the timing in relationship to the diagnosis of thyroid disease were recorded.

## Results

Of a total 333 children and adolescents with autoimmune thyroid disease, 9 (2.7%) were noted to have vitiligo (Tables 1 and 2). Eighty-seven had Graves' disease, and 4 of these patients with Graves' disease (4.6%) had vitiligo. Two hundred forty-six children had Hashimoto's thyroiditis, and 5 of these patients (2.0%) had vitiligo.

**Table 1:** Patients with Graves' Disease and Vitiligo

*Age of onset thyroid disease (yrs)*	*Age of onset vitiligo (yrs)*	*Gender*
3.5	Unknown (present at presentation)	M

3.5	Unknown (present at presentation)	F

4	4.5	F

5	Unknown (present at presentation)	M

**Table 2:** Patients with Hashimoto's Thyroiditis and Vitiligo

*Age of onset thyroid disease (yrs)*	*Gender*	*TSH* *(uU/ml)*
9.75	M	2

12	F	5.14

13	M	4.48

14.25	M	25

17.25	F	2.3

For the children with Graves' disease and vitiligo (Table 1), the average age of onset of thyroid disease was 4 ± 0.7 years (range 3.5 to 5 years). In 3 of the 4 children, vitiligo presented prior to the thyroid disease. One child developed vitiligo 6 months after Graves' disease was diagnosed. Males and females were represented equally among the patients with Graves' disease and vitiligo.

Of the patients with Hashimoto's thyroiditis and vitiligo (Table 2), all were clinically and biochemically euthyroid at the time of evaluation with the exception of one child with an elevated TSH level. Three of the 5 patients (60%) had positive anti-thyroid peroxidase (TPO) antibodies, and 2 (40%) had anti-thyroglobulin (Tg) antibodies. The average age at diagnosis of thyroid disease was 13.25 ± 2.8 years (range 9.75 to 17.25 years). Three of the children were males and 2 were females.

## Discussion

Studying a large cohort of children with Graves' disease and Hashimoto's thyroiditis, we find that 4.6% and 2.0% of children, respectively, have vitiligo. Interestingly, the children with vitiligo and Graves' disease were much younger than those with Hashimoto's disease and vitiligo.

Graves' disease is rare in the pediatric population, with a prevalence of about 1 per 10,000 individuals [12,13]. In children, the peak age of Graves' disease is 11-15 years, and it is three to five times more common in females than in males. In young children, no gender differences have been found [14,15].

When Hashimoto's thyroiditis occurs in pediatric patients, it is most common in adolescence, and is rare before the age of 3 [19]. The incidence of Hashimoto's in adolescence ranges from 1-2% of individuals [18-20]. However, an NHANES study found that 6.3% of adolescents from 12 to 19 years of age had positive anti-thyroglobulin antibodies and 4.8% had anti-thyroid peroxidase antibodies [16]. Another study of 160 children with antibodies consistent with euthyroid autoimmune thyroiditis had a mean age of 9.1 years [17].

As T-cell-mediated autoimmune diseases, Graves' disease and Hashimoto's thyroiditis have lymphocytic infiltration into thyroid parenchyma [10,14,19]. In Graves' disease, the antibodies bind to thyrotropin receptors, stimulating thyroid hormone production [10,14,19]. In Hashimoto's thyroiditis, lymphocytic infiltration leads to thyroid destruction [10]. Similarly, skin biopsies from vitiligo patients show dermal and epidermal lymphocytic infiltrate, consisting of activated T cells, which are thought to cause melanocyte destruction [8].

Vitiligo is the most common acquired pigmentary disorder in children and adults, with an incidence of approximately 1% in the general population [1-5]. Vitiligo results from loss of melanocytes, which leads to well-demarcated depigmentation in macules or patches on skin, overlying hair, and/or mucus membranes. Onset of vitiligo prior to age 20 years occurs in approximately 50% of cases and before 10 years in approximately 25%, with a nearly equal gender ratio [1,4-7]. Several etiologies for vitiligo have been proposed, and significant evidence supports an autoimmune pathogenesis, with circulating autoantibodies that target melanocyte antigens and subsequently attack and destroy melanocytes [1,3-5]. This theory is supported by the recent identification of genes linked to vitiligo that are involved in innate immunity [5,6].

Studies have shown an association between vitiligo and thyroid disease with an 8-25% incidence of autoimmune thyroid disease in patients with vitiligo [2,3,5,6]. Hashimoto's thyroiditis is seen in the majority of adult patients with vitiligo and autoimmune thyroid disease [2,3,22]. As in our cohort, vitiligo was diagnosed before thyroid disease in most patients in these studies [1,3].

The age of onset of vitiligo has been found to be earlier in families with a history of multiple autoimmune diseases [22]. This may be at least in part due to variants of genes including NALP1 that have been associated with susceptibility to vitiligo and autoimmune thyroid disease [11]. This gene regulates the immune system, including activating the inflammatory cascade, and is expressed on T cells and Langerhans' cells [11].

Studies have also identified immunogenetic associations with vitiligo and autoimmune thyroid diseases. Vitiligo is associated with HLA-DR4, and thyroid disease is associated with Class 1 and Class II HLA including HLA-DR [8-10]. Graves' disease has been associated with HLA-DR3 [9,14,20]. In comparison, Hashimoto's thyroiditis has not had a consistent HLA-association, but HLA-DR has been associated [9]. Other studies have shown an increase in CD4+ T-lymphocytes in addition to an elevated CD4+/CD8+ ratio in vitiligo patients [8], a finding that is present in patients with autoimmune thyroid disease [9,10].

Similarly, patients with vitiligo and at least one other autoimmune condition, including either Graves' disease or Hashimoto's thyroiditis, have been found to have a cytotoxic T lymphocyte antigen-4 (CTLA-4) polymorphism, which is involved in T cell apoptosis [8-10]. In Graves' disease, a specific CTLA-4 polymorphism has been associated with an early age of onset and severity of presentation [9]. Thus in the future, it will be interesting to characterize the genetic associations of vitiligo and other autoimmune diseases in the pediatric population.

Overall, our observations of a significant incidence of vitiligo in children with thyroid disease shows that children with thyroid disease should be screened for vitiligo, especially young children with Graves' disease. Thyroid screening is already recommended annually for patients with vitiligo [1-6]. The occurrence of Graves' disease and vitiligo in young children further supports the notion that the nature of the autoimmune disorder in the young population differs from that seen in older children.

## Competing interests

The authors declare that they have no competing interests.

## Authors' contributions

BP carried out the chart review and performed the data analysis. SR conceived of the study, and participated in its design and coordination. All authors read and approved the final manuscript.
